# Human NF-κB1 Haploinsufficiency and Epstein–Barr Virus-Induced Disease—Molecular Mechanisms and Consequences

**DOI:** 10.3389/fimmu.2017.01978

**Published:** 2018-01-18

**Authors:** Birgit Hoeger, Nina Kathrin Serwas, Kaan Boztug

**Affiliations:** ^1^Ludwig Boltzmann Institute for Rare and Undiagnosed Diseases, Vienna, Austria; ^2^CeMM Research Center for Molecular Medicine of the Austrian Academy of Sciences, Vienna, Austria; ^3^Department of Pediatrics and Adolescent Medicine, Medical University of Vienna, Vienna, Austria; ^4^Department of Pediatrics, St. Anna Kinderspital and Children’s Cancer Research Institute, Medical University of Vienna, Vienna, Austria

**Keywords:** nuclear factor kappa-light-chain-enhancer of activated B cells 1, haploinsufficiency, common variable immunodeficiency, combined immunodeficiency, Epstein–Barr virus, lymphoproliferation, T cells, B cells

## Abstract

Nuclear factor kappa-light-chain-enhancer of activated B cells 1 (NF-κB1)-related human primary immune deficiencies have initially been characterized as defining a subgroup of common variable immunodeficiencies (CVIDs), representing intrinsic B-cell disorders with antibody deficiency and recurrent infections of various kind. Recent evidence indicates that NF-κB1 haploinsufficiency underlies a variable type of combined immunodeficiency (CID) affecting both B and T lymphocyte compartments, with a broadened spectrum of disease manifestations, including Epstein–Barr virus (EBV)-induced lymphoproliferative disease and immediate life-threatening consequences. As part of this review series focused on EBV-related primary immunodeficiencies, we discuss the current clinical and molecular understanding of monoallelic *NFKB1* germline mutations with special focus on the emerging context of EBV-associated disease. We outline mechanistic implications of dysfunctional NF-κB1 in B and T cells and discuss the fatal relation of impaired T-cell function with the inability to clear EBV infections. Finally, we compare common and suggested treatment angles in the context of this complex disease.

## Introduction

Nuclear factor kappa-light-chain-enhancer of activated B cells (NF-κB) represents a key node in propagation of cellular signals, driving cell fate decisions toward proliferation, or apoptotic clearance. Due to their highly adaptable nature, immune cells in particular rely on NF-κB processes for their development, function and inflammatory responses. Both the innate and adaptive immune axes are critically dependent on functional NF-κB signaling networks (1). Owing to its name, NF-κB-derived programming of target genes particularly manages B-cell fates including their maturation, survival, differentiation and (T-cell independent) class switching processes (2, 3). Its importance for maintaining B-cell development and integrity has been underlined by studying murine knockout models (4) and the identification of human *NFKB1* and *NFKB2* gene defects with B-cell deficiency-related clinical manifestations initially classified as common variable immunodeficiencies (CVIDs) (5–18). Beyond its role in B-cell intrinsic processes, NF-κB1 defects presenting with recurrent or chronic Epstein–Barr virus (EBV) infection (6, 8) or fatal EBV-driven lymphoproliferative disease (7) suggest a broadened phenotypic spectrum, including combined immunodeficiency (CID) with B- and T-cell dysfunction (7). In addition, NF-κB1 has been shown to regulate human NK-cell maturation and effector function *in vitro*, with yet unclear consequences for human health (13). Together, these studies highlight the relevance of identifying human genetic defects for studying key processes in immune cell function and deciphering the spectrum of phenotypic consequences of newly emerging disorders. In this review article, we focus on discussing EBV-associated disease in the context of genetic predisposition to NF-κB1 dysfunction. A general review of NF-κB mechanisms in and beyond B cells and EBV pathogenesis has been discussed elsewhere (1–3, 19, 20) and is outlined here in comparing B- and T-cell functions related to the emergence of NF-κB-related EBV infectious disease.

## NF-κB Signaling

**Figure 1:**
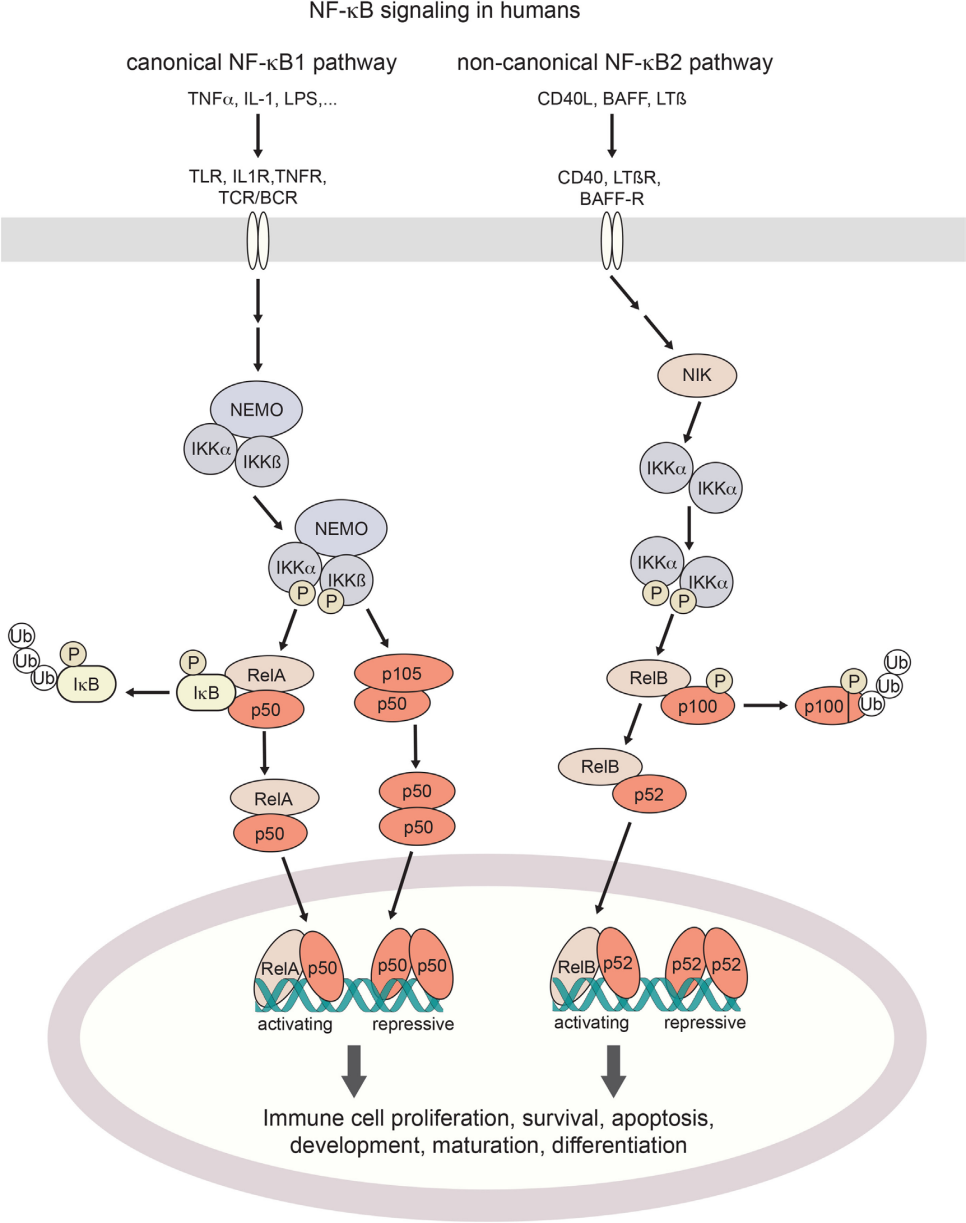
Canonical and non-canonical NF-κB signaling in humans. Activation of the canonical NF-κB pathway is triggered by a broad range of proinflammatory cytokines such as TNFα or IL-1, bacterial pattern recognition molecules such as LPS, or antigen stimulation. Non-canonical signaling is triggered by TNF family receptors and their ligands, resulting in activation of NIK kinase activity. Both pathways cumulate in the activation of IKK (IκB-kinases) which phosphorylate inhibitory IκB binding partners for their poly-ubiquitination and proteosomal degradation (canonical axis) or the processing of p100 into its active form (non-canonical axis). Resulting NF-κB dimers translocate to the nucleus. Depending on their assembly into activating hetero- or repressive homo-dimeric conformations, NF-κB signaling regulates the expression of hundreds of target genes. TNF(R), tumor necrosis factor (receptor); IL-1(R), interleukin-1 (receptor); LPS, lipopolysaccharide; BAFF(-R), B-cell activating factor (receptor); LTβ(R), lymphotoxin β (receptor); TLR, toll-like receptor; TCR/BCR, T-cell/B-cell receptor; NIK, NF-κB inducing kinase; NEMO, NF-κB essential modulator; IKK, IκB kinase; IκB, Inhibitor of NF-κB; NF-κB, nuclear factor kappa-light-chain-enhancer of activated B cells.

Proteins of the NF-κB family of transcription factors are broadly expressed in cells of the immune system. The family consists of the two large precursor proteins NF-κB1 (p105) and NF-κB2 (p100), which can be processed to their smaller mature subunits p50 and p52, respectively, and their dimerization partners RelA (p65), RelB, and c-Rel (21). Upon NF-κB pathway activation, p105 and p100 are post-translationally processed for nuclear translocation of the resulting mature NF-κB dimers (22) (Figure 1). In resting state, NF-κB shows cytoplasmic localization. This is achieved through masking the nuclear localization sequence, either by binding of inhibitor of NF-κB (IκB) proteins to p50 dimers, or through *in-cis* binding of the C-terminal part of p105 (23).

Activation of the NF-κB pathway occurs through two distinct routes, the canonical and the non-canonical pathway, by distinct stimuli-receptor pairs (21, 24) as described in Figure 1. Specifically, in canonical signaling, binding of TNFα to TNF receptors results in the recruitment of the adaptor protein TRADD to the intracellular part of the TNFR which in turn activates the kinase RIP1 and subsequently IKK (25, 26). IL-1 or LPS binding to their respective receptors (IL-1 receptors and Toll-like receptors, respectively) results in the recruitment of adaptor proteins MyD88 and TRIF to the TIR (TLR/interleukin-1 receptor) domain. This induces IRAK1/4-dependent recruitment of IKK to the complex, and its subsequent activation through TRAF6 (27, 28). BCR and TCR signaling also result in the activation of IKK, which happens through the recruitment of the CARMA, BCL10, and MALT1 complex by PKCβ in B cells and PKCθ in T cells (29, 30). Stimulation of IKK (IκB-kinase)-α, IKKβ, and NEMO (IKKγ) induces phosphorylation and degradation of IκB (inhibitor of κB) proteins (23), with subsequent release of NF-κB dimers for nuclear translocation. By contrast, non-canonical NF-κB signaling induced by TNF receptor family ligands CD40L, BAFF or lymphotoxin-β (24) inhibits TRAF3-induced NIK degradation, leading to NIK kinase-dependent activation of primarily IKKα (31). Activated IKKα in turn phosphorylates NF-κB2/p100 which results in processing to its active form p52, and its nuclear translocation (32). Target genes are modulated by either activating or repressing interactions with κB DNA-binding sites (33). As the transactivation domain is provided by the Rel binding partners, NF-κB homodimers exert a repressive function. Substantial crosstalk between the canonical and non-canonical NF-κB signaling axes has been uncovered, forming the basis for a complex and tightly regulated signaling network that shapes cell-type and cell-state-specific functions (34).

In the modulation of immune-relevant processes, transcriptional activity of NF-κB is required during negative selection in T-cell development. This has been elucidated through the analysis of *Relb^−/−^* mice which develop spontaneous autoimmune dermatitis (35). Furthermore, NF-κB-mediated transcription is important for the development and maturation of NK and NKT cells in mice (36, 37). Most importantly, NF-κB transcriptional activity is required during B-cell development, maturation and survival. Already at the pre-BCR stage in B-cell development, NF-κB provides pro-survival signals (38). Deletion of IKK specifically from B cells results in reduced numbers of transitional and mature B cells in mice (39). Similarly, primary immunodeficiency (PID) diseases caused by mutations in the genes encoding family or pathway members of NF-κB predominantly present with B-cell deficiencies, as discussed below.

## Human Germline Mutations in *NFKB1* and Their Consequences in B- and T-Cell Function

### NF-κB1 Defects with CVID-Like Presentation

Common variable immunodeficiency denotes a heterogeneous group of B-cell disorders characterized by pronounced antibody deficiency and recurrent infections. It is considered the most prevalent symptomatic PID for which an underlying monogenic origin has been identified in only ~10% of cases (40–42). The first clinical manifestations of NF-κB1 haploinsufficiency and CVID-like presentation were reported in 2015 (5). Ever since, further cases of *NFKB1* defects with monoallelic inheritance have been reported (6–13), broadening our understanding of NF-κB1 as critical factor in B-cell function. As appreciated from murine studies, mice deficient in NF-κB were shown defective in B-cell maturation, isotype switching, class-switch recombination, antibody response, and humoral immune response with increased susceptibility to infection, especially of the *S. pneumoniae* type (4, 43–46). T cells have been reported with abnormal proliferation responses. Intriguingly, development and reproduction of *Nfkb1^−/−^* mice is unaffected (43).

The initial report on NF-κB1 haploinsufficiency in humans describes three unrelated kindreds with 20 affected individuals presenting with CVID and/or hypogammaglobulinemia (5). Subsequent studies on patients haploinsufficient for NF-κB1 have reported an extended clinical phenotype and a predominant CVID-like presentation (6–13). Among the affected individuals, clinical manifestations were highly variable as summarized in Table 1. Immunoglobulins were mildly to severely decreased, and infections ranged from common sinusitis to progressive pulmonary disease. Autoimmune episodes were evident in some individuals, including autoimmune hypothyroidism, autoimmune anemia, cytopenias, and/or splenomegaly. Partly severe autoinflammatory conditions were apparent in a fraction of described patients (Table 1). A recent study on the consequences of NF-κB1 haploinsufficiency on the B-cell compartment revealed low numbers of peripheral B cells with normal T-cell counts, low numbers of CD27^+^ switched-memory B cells and an expansion of CD21^lo^ B cells (6). Furthermore, an impairment of early stages of B-cell differentiation upon monoallelic loss of *NFKB1* was detected, with partial maturational arrest at the pre-BI stage (6). Investigation of T-cell compositions in another study reported low CD4^+^ effector memory T-cell as well as T_h_17 memory subsets in the respective individuals (9). Schipp and coworkers have observed decreased naïve CD4 and memory T-cell states, and increased double-negative T cells for an affected individual, with defective FasL-mediated apoptosis (8).

**Table 1:** Clinical presentation of monoallelic loss of nuclear factor kappa-light-chain-enhancer of activated B cells 1 (NF-κB1) function. A comparison of phenotypes of affected individuals shows various occurrences and degrees of infections, autoimmunity and autoinflammatory syndromes, and varying manifestations of Epstein–Barr virus (EBV). Genetic information, diagnosis and clinical symptoms were extracted from the respective studies. Individuals are listed according to study reference, respective patient code, and type of mutation. Information about treatment and surgery is shown, if indicated in the respective publications. Asymptomatic mutation carriers are not listed.

Reference	Patient	G	Mutation	AoO	Diagn.	Symptoms			IgG (g/L)	IgA	IgM	Treatment	Surgery
						Infection	Autoimmunity/Autoinflammation	Other					
(5)	FamNL1_16	♀	c.730+4A>Gp.D191_K244delinsE	29 years	Common variable immunodeficiency (CVID)	Respiratory tract infections, pansinusitis, atrophic gastritis, pneumonia	COPD	Pyoderma gangrenosum, squamous cell carcinoma, pulmonary insufficiency	2.76	0.30	0.16	IVIG, etanercept	n/a

	FamNL1_18	♀	c.730+4A>Gp.D191_K244delinsE	65 years	CVID	Sinopulmonary infections, *Campylobacter* enteritis	Splenomegaly, lymphadenopathy	Ischemic heart disease, lung adenocarcinoma, thrombocytopenia	2.49	0.13	0.53	n/a	n/a

	FamNL1_19	♀	n/a	46 years	–	Bronchitis, pneumonias (*H. influenzae*)	COPD	Coronary insufficiency, impaired pulmonary function	3.0	0.4	0.4	IVIG	n/a

	FamNL1_21	*♂*	c.730+4A>Gp.D191_K244 delinsE	31 years	CVID	pneumonias, recurrent sinusitis, bronchiectasis	COPD	Lung fibrosis, respiratory insufficiency	6.0	1.26	0.4	IVIG	Appendectomy

	FamNL1_23	♀	n/a	n/a	–	Recurrent lower airway infections	COPD	Defective vaccination response	HGG	n/a	n/a	IVIG	n/a

	FamNL1_24	♀	n/a	n/a		Recurrent lower airway infections	COPD with pulmonary insufficiency	Defective vaccination response, mannose-binding lectin deficiency	HGG	n/a	n/a	IVIG	n/a

	FamNL1_25	♀	c.730+4A>Gp.D191_K244 delinsE	52 years	CVID	Pulmonary infection	COPD	Respiratory insufficiency, pulmonary hypertension	4.6	1.0	0.4	IVIG	n/a

	FamNL1_34	*♂*	c.730+4A>Gp.D191_K244 delinsE	44 years	CVID	Recurrent respiratory infections	Hypothyroidism	Alopecia areata, pyoderma gangrenosum	8.0	0.83	0.25	IVIG	Surgery for VSD

	FamNL1_36 [P.2 (6), P.4 (13)]	♀	c.730+4A>Gp.D191_K244 delinsE	30 years	CVID	Pneumonia, recurrent sinusitis, otitis media, *Salmonella* enteritis	–	–	1.81	0.06	0.48	IVIG	n/a

	FamNL1_40	♀	c.730+4A>Gp.D191_K244 delinsE	34 years	CVID	Recurrent upper airway infection, sporadic lower respiratory tract infections	COPD	–	5.1	<0.5	0.65	IVIG	n/a

	FamNL1_42	♀	c.730+4A>Gp.D191_K244 delinsE	n/a	HGG	Recurrent paronychia, superficial skin infections	–	–	n/a	n/a	n/a	n/a	n/a

	FamNL1_48	♀	c.730+4A>Gp.D191_K244 delinsE	15 years	HGG	Folliculitis, furunculosis	Thyroiditis	Aortic stenosis	3.72	0.59	0.74	Levothyroxine	n/a

	FamNL1_49	♀	c.730+4A>Gp.D191_K244 delinsE	9 months	CVID	Giardiasis, recurrent respiratory tract infections, trichophyton skin infections	Thyroiditis	–	9.1^a^	1.41	0.11	IVIG	n/a

	FamNL1_57	♀	c.730+4A>G p.D191_K244 delinsE	39 years	CVID	Lymphocytic interstitial pneumonitis, recurrent sinopulmonary infections	–	Nodular regenerative hyperplasia, ongoing diarrhea, reticunodular and lymphocytic infiltrates, liver eosinophilic and lymphocytic infiltrates	1.3	<0.04	0.3	IVIG, IV-antibiotics, ursodeoxycholic acid	n/a

	FamNL1_62	♀	c.730+4A>Gp.D191_K244 delinsE	30 years	CVID	Recurrent sinus infections	–	–	4.7	0.4	0.3	IVIG	n/a

	Fam089_I1	*♂*	c.835+2T>Gp.K244_D279delinsN	64 years	CVID	Severe pneumonia with pleural empyema	–	Multiple liver hemangiomas	1.42	0.08	0.4	IVIG	n/a

	Fam089_II2 [P.5 (13)]	♀	c.835+2T>Gp.K244_D279delinsN	16 years	CVID	Recurrent pulmonary infections, pneumonia	Hemolytic anemia, hepatomegaly, lymphadenopathy	Idiopathic thrombocytopenic purpura, infection-induced neutropenia, intermittent diarrhea, recurrent arthralgias	6.29	<0.3	<0.3	IVIG, rhG-CSF	n/a

	Fam089_III2	♀	c.835+2T>Gp.K244_D279delinsN	14 months	HGG	–	–	Transient hypogammaglobulinemia	1.94	<0.07	0.29	n/a	n/a

	FamNZ_I2	♀	c.465dupAp.A156Sfs*12	n/a	–	–	–	Alopecia, thrombocytopenia	9.9	0.58	0.8	n/a	n/a

	FamNZ_II1	*♂*	c.465dupAp.A156Sfs*12	2 years	CVID	–	Hemolytic anemia	Thrombocytopenia, neutropenia	5.17	0.07	0.53	IVIG	Splenectomy

	FamNZ_II2	♀	c.465dupAp.A156Sfs*12	20 years	CVID	Bronchiectasis, recurrent infections	–	Alopecia, marginal zone non-Hodgkin lymphoma, nodular regenerative hyperplasia	n/a	n/a	n/a	Rituximab, chlorambucil, IVIG	n/a

(6)	Patient 1	*♂*	c.1517delC p.A506Vfs*17	7 years	CVID	Recurrent respiratory infections, pneumonia, sinusitis	Thyroiditis, enteropathy	Gastric adenoma	1.6	0.14	0.14	IVIG, budesonide	n/a

(7)	Index patient	♀	c.494delGp.G165Afs*32	2 years	CID with EBV-LPD	Recurrent respiratory infections, recurrent EBV infection with EBV-LPD	Hepatosplenomegaly, esophagitis, cervical/anxillary/supraclavicular lymphadenopathy	Bacterial parapharyngeal abscess, chronic abscess-forming inflammation, neutropenia, leukopenia, thrombocytopenia	2.32	<0.05	0.012	IVIG, antibiotics, corticosteroids, rituximab	Tonsillectomy, adenectomy

	Father	*♂*	c.494delGp.G165Afs*32	n/a	–	Non-respiratory tract infections	–	–	4.09	0.23	0.41	–	n/a

(8)	Patient 1	♀	c.139delAp.I47Yfs*2	n/a	CVID	Recurrent pneumonias, upper respiratory tract infection, fever, chronic otitis, mastoiditis, sinusitis, vulvovaginitis; recurrent viral (chronic EBV, recurrent herpes zoster, HPV38, HSV, RSV), bacterial (*Gardnerella* and group *A Streptococcus, C. braakii)*, Candida (C. *lusitaniae*) infections	Hemolytic anemia, idiopathic thrombocytopenia, pancytopenia, autoantibodies, organ infiltration (liver, lung, spleen, kidney) with hepatosplenomegaly, pancolitis, generalized mucositis, recurrent aphthae and painful ulcers of mouth, esophagus, and genitalia	Recurrent idiopathic diarrhea, abdominal pain, bloody stools, ascites, intermittent proteinuria, latent hypothyreosis, multiple ovarian cysts, lack of calcium and vitamin D, recurrent headaches with vertigo, numbness and paresis of left arm and hand	4.85	<0.06	0.24	Steroids, mycophenolate, IVIG, mofetil, antibiotics, antiviral medication, calcium, vitamin D	Cholecystectomy, colectomy, mastoidectomy, ulcer excision, myringotomy, tympanostomy

	Patient 2	♀	c.469C>T p.R157*	n/a	CVID	Recurrent pneumonias *(S. pneumoniae)*, upper respiratory tract infection, *H. influenzae, Salmonella* infections	Hemolytic anemia, idiopathic thrombocytopenia, leukopenia, splenomegaly	Recurrent diarrhea	10.9	<0.05	3.62	IVIG, steroids	n/a

(9)	F1.II-4	♀	c.200A>G p.H67R	n/a	Behçet-like; AB def	Upper respiratory tract infection	Recurrent monoarthritis, complex aphthae (mouth, genitalia), small vessel vasculitis	Periodic abdominal pain, chronic idiopathic diarrhea	HGG	n/a	n/a	IVIG	n/a

	F1.III-2	♀	c.200A>G p.H67R	n/a	Behçet-like; AB def	–	–	Benign kidney tumor	HGG	n/a	n/a	n/a	n/a

	F1.III-3	*♂*	c.200A>G p.H67R	n/a	Behçet-like; AB def	Upper respiratory tract infection	Complex aphthae (esophagus)	Rudimentary left kidney, febrile attacks	HGG	n/a	n/a	IVIG	n/a

	F1.III-6	♀	c.200A>G p.H67R	n/a	Behçet-like; AB def	Upper respiratory tract infection	Complex aphthae (mouth)	–	HGG	n/a	n/a	IVIG	n/a

	F1.III-7	♀	c.200A>G p.H67R	n/a	Behçet-like	Upper respiratory tract infection	Recurrent monoarthritis, hyperinflammatory response to tooth excision, complex aphthae (mouth, genitalia)	Febrile attacks, periodic abdominal pain, keratitis	n/a	n/a	n/a	n/a	n/a

	F1.III-8	♀	c.200A>G p.H67R	n/a	Behçet-like; AB def	Upper respiratory tract infection	Recurrent monoarthritis, microscopic colitis, complex aphthae (mouth)	Febrile attacks, periodic abdominal pain	HGG	n/a	n/a	IVIG	n/a

	F1.IV-2	♀	c.200A>G p.H67R	n/a	Behçet-like; AB def	Upper respiratory tract infection	Complex aphthae (mouth, genitalia)	Febrile attacks, periodic abdominal pain	HGG	n/a	n/a	n/a	n/a

	F2.II-3	♀	c.1659C>Gp.I553M	n/a	CVID	Respiratory tract infection, autoimmune hypothyroiditis	Spondyloarthropathy oligoarthritis	Chronic idiopathic diarrhea, asthma	HGG	n/a	n/a	IVIG	n/a

	F2.III-2	*♂*	c.1659C>Gp.I553M	n/a	CVID	Respiratory tract infection	Celiac disease, asthma	–	n/a	n/a	n/a	n/a	n/a

	F3.II-1	*♂*	c.469C>Tp.R157*	n/a	FNC	Respiratory tract infection	Postoperative necrotizing cellulitis	–	n/a	n/a	n/a	n/a	Yes (unknown)

	F3.II-5	*♂*	c.469C>Tp.R157*	n/a	FNC	–	Postoperative necrotizing cellulitis	–	n/a	n/a	n/a	n/a	Yes (unknown)

(10)	Patient 1	*♂*	c.1301-1G>A(intronic)	42 years	CVID	Pneumonias, chronic sinusitis, conjunctivitis, otitis, shingles	Lymphoid hyperplasia, aphthous ulcers, hypersplenism	Lung granulomas, enteropathy, neutropenia	Tx	<0.07	<0.11	n/a	Splenectomy

	Patient 2	♀	c.1301-1G>A (intronic)	19 years	CVID	Pneumonias, chronic sinusitis, conjunctivitis, otitis, shingles, *C. difficile* colitis	–	Morphea	<0.51	<0.05	<0.05	n/a	n/a

	Patient 3	*♂*	c.259-4A>G (intronic)	21 years	CVID	Pneumonias, empyema, chronic sinusitis	Bronchiectasis, hypothyroidism, vitiligo	–	Tx	<0.01	<0.05	n/a	Lobectomy

	Patient 4	♀	c.957T>A p.Y319*	19 years	CVID	MAl, pneumonia, lung abscesses, PML	Autoimmune hemolytic anemia, immune thrombocytopenia	Aplastic bone marrow	Tx	<0.07	<0.l7	n/a	Splenectomy

	Patient 5	♀	c.1375delT p.F459Lfs*26	7 years	CVID	Pneumonias, otitis, giardiasis, *C. difficile* colitis, HSV infection, cellulitis, MAI	Bronchiectasis	Enteropathy, osteopenia, poor growth	Tx	<0.02	<0.02	n/a	n/a

(11)	Patient 26	♀	n/a	n/a	CVID	n/a	n/a	n/a	n/a	n/a	n/a	n/a	n/a

	Patient 27 [P.6 (13)]	*♂*	c.469C>T p.R157*	n/a	CVID	n/a	n/a	n/a	n/a	n/a	n/a	n/a	n/a

	Patient 28	♀	n/a	n/a	CVID	n/a	n/a	n/a	n/a	n/a	n/a	n/a	n/a

(12)	P9.1		c.904dupT p.S302Ffs*7	n/a	AB def	*H. influenzae*, pulmonary fibrosis	Autoimmune hemolytic anemia	–	n/a	n/a	n/a	Prednisol., rituximab	n/a

	P9.2		c.904dupT p.S302Ffs*7	n/a	AB def		Autoimmune hemolytic anemia, immune thrombocytopenia, autoimmune neutropenia	–	n/a	n/a	n/a	Prednisol., Ig	n/a

(13)	Patient 1	*♂*	c.1517delC p.A506Vfs*17	13 years	CVID	Recurrent pulmonary infections	Autoimmune thyroiditis, autoimmune enteropathy	Gastric adenoma	1.6	0.1	0.1	n/a	n/a

	Patient 2	♀	c.1365delT p.V456*	33 years	CVID	Pneumonias, necrotizing tonsillitis, periodontitis, infections (h. zoster, CMV viremia, intermittent low-grade EBV)	Autoimmune cytopenia, splenomegaly, lymphadenopathy, interstitial lung disease	Multiple liver hemangiomas	6.7	0.3	0.3	n/a	n/a

	Patient 3	*♂*	c.1365delT p.V456*	43 years	CVID	Recurrent sinusitis and otitis, pneumonia, *Salmonella* enteritis	Autoimmune cytopenia, arthritis, splenomegaly, vitiligo, lymphadenopathy, granulomatous lung disease	–	0.08	0.05	0.05	n/a	n/a

	Patient 6 [p.27 (11)]	*♂*	c.469C>T p.R157*	47 years	CVID	Chronic sinusitis, recurrent otitis, pneumonia, JC virus encephalitis, norovirus, EBV reactivation	Skin abscesses, atopic dermatitis, autoimmune enteropathy, nodular regenerative hyperplasia, splenomegaly, lymphadenopathy, thrombocytopenia	–	2.7	<0.06	0.21	n/a	n/a

	Patient 7	♀	c.295C>T p.Q99*	20 years	CVID	Recurrent bronchitis, sinusitis	Enteropathy, splenomegaly	Basal cell carcinoma, osteoporosis	n/a	n/a	n/a	n/a	n/a

*G, gender; AoO, Age of onset (diagnosis); Diagn., diagnosis; COPD, chronic obstructive pulmonary disease; IVIG, intravenous immunoglobulin; HGG, hypogammaglobulinemia; VSD, ventricular septal defect; EBV-LPD, Epstein–Barr virus-associated lymphoproliferative disease; AB def, antibody deficiency; FNC, familial necrotizing cellulitis; prednisol., prednisolone; MAI, mycobacterium avium intracellulare; PML, progressive multifocal leukoencephalopathy; n/a, not applicable*.

^a^*After IVIG*.

Overall, monoallelic mutations in *NFKB1* have been shown to impose B-cell dysfunction including immunoglobulin and antibody deficiencies often accompanied by autoimmune and also autoinflammatory responses. Detailed investigations of B-cell differentiation and function have been initiated based on the reported findings, and would benefit from further studies in human cell systems. As generally seen with monoallelic and especially haploinsufficient immunodeficiencies, penetrance of disease manifestation is below 100% and age of onset is variable in individuals with *NFKB1* mutations (5).

### NF-κB1 Haploinsufficiency and Predisposition to EBV Infection and EBV-Lymphoproliferative Disease

The first notions of importance of NF-κB1 signaling in defense against EBV in humans were published in 2016. Schipp and coworkers reported a patient with a novel *NFKB1* frame-shift mutation that presented with chronic EBV infection in context of a complex and severe multi-system CVID-like disease (8). In 2017, two additional CVID patients with heterozygous *NFKB1* loss were reported with low-grade or reactivating EBV (see Table 1) (13).

Recently, we reported a novel case of NF-κB1 haploinsufficiency broadening the spectrum of disease manifestation by T-cell defects with severe EBV-associated lymphoproliferative disease (7). The patient presented with recurrent infections, autoimmunity manifestation, and with two severe episodes of EBV-associated lymphoproliferative disease (Table 1). In addition to low CD19^+^ B cells, reduced non-switched and switched memory B cells and low immunoglobulin levels, T-cell proliferation was impaired. Referring to NF-κB1 haploinsufficiency primarily known as B-cell disorder, the severe EBV-lymphoproliferative episodes were interpreted as a new feature of this disease and attributed to the apparent T-cell dysfunction (7).

Interestingly, EBV infections have not been reported among the common *NFKB2* mutations with functional p52 haploinsufficiency causing CVID (12, 16–18, 47–49). By contrast, an NF-κB2 mutant (p.R635*) with constitutive nuclear localization has been shown associated with multiple infections, including EBV viremia in one of three affected individuals (14).

### Consequences of *NFKB1* Mutations on Protein Function

**Figure 2:**
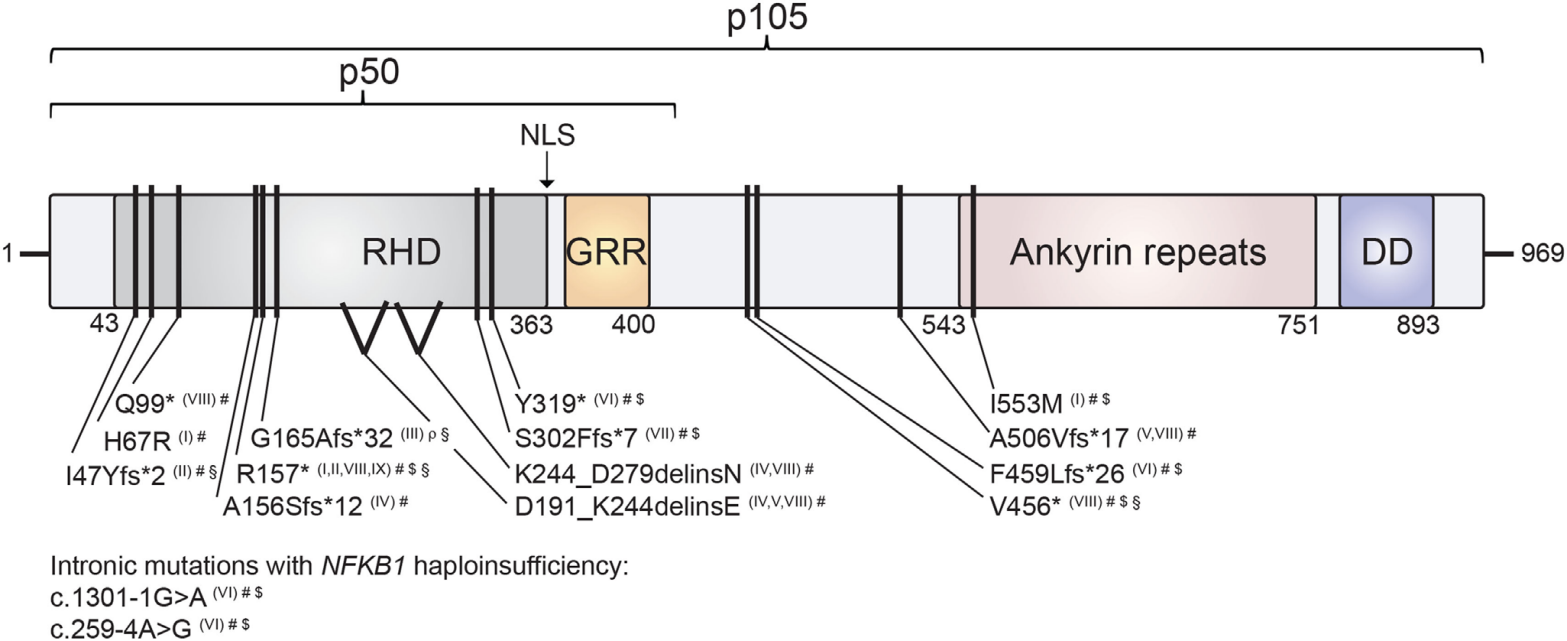
Mapping of human nuclear factor kappa-light-chain enhancer of activated B cells 1 (NF-κB1) mutations on the protein’s domain architecture. Comparison of identified NF-κB1 mutations and their clinical presentation reveals no obvious genotype–phenotype correlation. Individual mutations in either p105 or p105/p50 protein subunits of NF-κB1 are depicted by black bars. Stop codons are depicted by asterisks. NF-κB1 haploinsufficiency results from intronic mutations, non-sense mutations and frameshift mutations with premature truncations. Similarly, two identified splice-site mutations result in NF-κB1 haploinsufficiency due to loss of mutated precursors. Missense exchange p.I553M results in enhanced degradation of both p105 and p50, while p.H67R leads to reduced nuclear entry of the affected mutant p50 and, hence, to functional haploinsufficiency. NLS, nuclear localization sequence; RHD, Rel homology domain; GRR, glycine-rich region; DD, death domain. NF-κB1 mutations are referenced according to Roman numbering: (I) Kaustio et al. (9); (II) Schipp et al. (8); (III) Boztug et al. (7); (IV) Fliegauf et al. (5); (V) Lougaris, Moratto et al. (6); (VI) Maffucci et al. (10); (VII) Rae et al. (12); (VIII) Lougaris, Patrizi et al. (13) (IX) Keller et al. (11). Clinical presentations are indicated by the following symbols: #, common variable immunodeficiencies (CVID) (incl. autoimmunity); $, autoinflammation; §, chronic EBV/EBV-lymphoproliferation; ρ, combined immunodeficiency (CID).

The comparison of the consequences of the various *NFKB1* mutations for protein integrity and function represents a first-line consideration of similarities and differences of NF-κB1-related disease manifestations. The distinct disease-associated monoallelic mutations in *NFKB1* that have been identified to date (6–13) span intronic and exonic alterations, non-sense mutations, splice-site donors and frameshift mutations, all resulting in NF-κB1 haploinsufficiency, as well as heterozygous missense mutations with functional haploinsufficiency (Table 1). Mapping these within the genetic locus and within the NF-κB1 protein domains depicts an accumulation of mutations especially in the Rel-homology domain (RHD) of p105/p50 (Figure 2). This domain is responsible for dimerization and DNA-binding abilities of the mature p50 subunit. This local accumulation includes a frameshift mutation with premature termination at position p.G165Afs*32 in the central part of the RHD, which has been associated with EBV lymphoproliferative disease (7), as well as further EBV-associated mutations in close proximity (p.R157*, p.I47Yfs*2) (8, 13) (Figure 2). Still, other mutations introducing early stop codons have been identified in the RHD domain lacking an association with EBV infection (5, 6, 9, 10, 12). In addition, mutation p.R157* that has been reported with EBV reactivation episodes (13) has been identified in unrelated patients without apparent EBV infection (8, 9, 11). Similarly, other clinical manifestations such as autoinflammatory syndromes do not correlate with clustering of mutations on protein domains (Figure 2). In conclusion, a rational genotype–phenotype relationship between NF-κB1-related disease manifestations cannot be found. Even though to date more than 55 *NFKB1* germline mutation carriers have been identified, larger case series should be established to perform meaningful phenotypic correlation studies. Also, the presence of modifying factors, such as secondary mutations and epigenetic alterations, could be addressed in such enlarged cohorts.

## Molecular Mechanisms of NF-κB1 Defects and EBV-Induced Disease

Epstein–Barr virus infections pose a huge challenge to immunocompromised individuals, given the virus’ wide prevalence in the adult population and its concomitant risk to induce life-threatening lymphoproliferative and malignant disease (50). EBV belonging to the herpesvirus family, was the first human tumor virus associated with B-cell and T-cell lymphoproliferative disease and related lymphoma (20, 50). Its mechanisms and implications for human health have been discussed by numerous studies [see, for example, reviews authored by Hatton and coworkers (19) or Thorley-Lawson (20)]. EBV primarily infects B cells and drives their proliferation by expressing a small number of latency genes that mimic growth, transcription, and anti-apoptotic markers, followed by a lytic replication phase to produce infectious virus (20). Successfully cleared by cytotoxic T and NK cells that interfere with different stages of infected B cells in healthy individuals (20, 51–53), immunodeficient humans fail to manage and eliminate these. As a consequence of cytotoxic T-cell dysfunction, accumulation of EBV-infected autoreactive B cells in target organs induces a concomitant infiltration of autoreactive T cells that results in harsh autoimmune episodes (54). EBV-associated disease is, thus, a phenomenon of infected B cells but their compromised elimination results from mainly T-cell intrinsic mechanisms.

As recent cases of NF-κB1 haploinsufficiency have been shown associated with EBV infection (8, 13) and EBV-lymphoproliferative disease with T-cell dysfunction (7), we compare NF-κB1 haploinsufficiency with other EBV-associated PIDs, discuss combined immunodeficiencies related to NF-κB by shared signaling pathways but without sign of EBV-associated disease, and outline the consequences of NF-κB1 dysfunction for T-cell integrity resulting in a proposed impairment of EBV clearance.

### Comparison of EBV-Associated PIDs and What to Learn from Them

Epstein–Barr virus-driven lymphoproliferative disease is commonly understood as consequence of impaired cytotoxic T- or NK-cell function. Several PIDs have been clearly associated with EBV-induced disease and reviewed on various occasions (55–59). The investigation of how these PIDs disturb T-cell function to affect virus elimination abilities is of critical relevance for understanding disease mechanisms.

Among the PIDs predisposing to severe infections, some predispose to a single, while others respond to a multitude of pathogens (60, 61). Specific EBV-associated disease has been reported for X-linked lymphoproliferative syndrome (XLP)-1 and -2 [BIRC4 and SAP deficiency, respectively (62, 63)], ITK (64), CD27 (65), CD70 (66, 67), CORO1A (68), RASGRP1 (69), and MAGT1 (X-MEN syndrome) (70) deficiencies, as summarized by various studies (55, 56, 59, 71). Clinically, these manifest in CID with antibody deficiencies and autoimmune episodes, including lymphoproliferative disease (55). Among the PIDs associated with risk of infections of various kinds, mutations of ATM, WASP, STK4, PIK3CD, PIK3R1, CTPS1, CARD11, FCGR3A, MCM4, and GATA2 have been reported with severe EBV infections (59).

Although impaired B- and NK-cell function were shown involved in EBV-associated diseases, it is primarily the cytotoxic T cells that initiate an effective—or defective—EBV-directed immune response (57, 60, 61, 71). A related disease state manifests through either a loss of effective T cells in the circulation, or through defective T-cell function (57).

**Figure 3:**
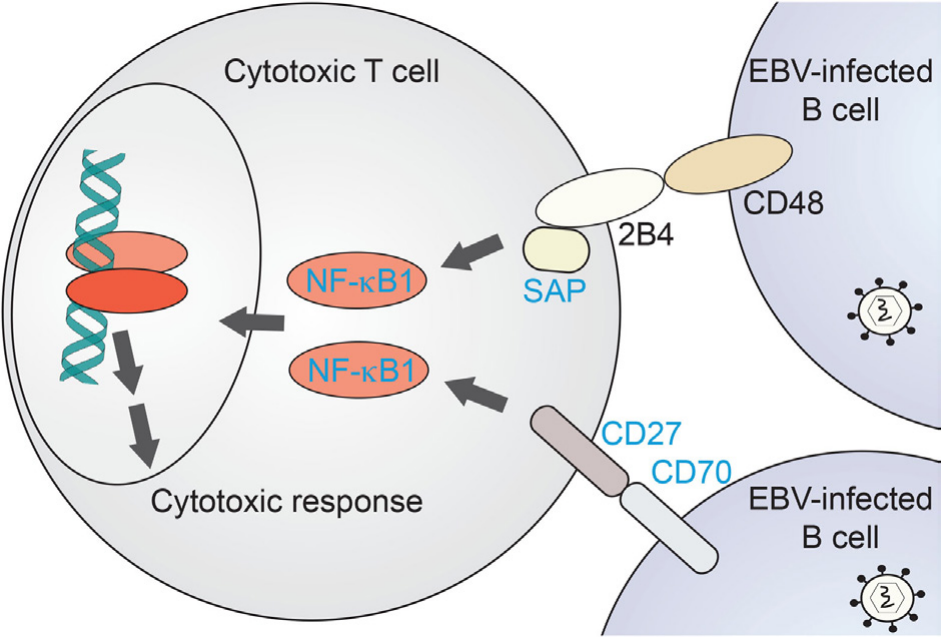
NF-κB1-related immunodeficiencies affecting T-cell mediated clearance of EBV-infected B cells. Proposed scenario for NF-κB1 signaling induced in cytotoxic T cells downstream of B-cell recognition events. Proteins for which primary immunodeficiencies (PIDs) with EBV-associated disease have been identified are named in blue. NF-κB1, nuclear factor kappa-light-chain enhancer of activated B cells-1; SAP, SLAM-associated protein.

NF-κB1 plays a critical role in cytotoxic T-cell function. A comparison with EBV disease-associated proteins and their canonical pathways underlines the importance of NF-κB1 signaling in recognition of EBV-infected B cells and their targeting for elimination. As recently reviewed by Tangye and coworkers (55), defined EBV-associated PID genes do share common pathways upstream of NF-κB1 in this specific process. NF-κB1 has been shown a relevant effector of B-cell recognition by cytotoxic T cells (Figure 3). CD70, a surface marker on (EBV-infected) B cells, binds to the CD27 receptor on T cells which triggers an NF-κB1-mediated response for expansion of cytotoxic T cells directed against the infected target cells (66, 67, 72). Loss of both CD70 and CD27 have been reported with CID-like syndromes, including EBV-driven malignancy and life-threatening EBV infection, respectively (65–67). Similarly, CD48/2B4 receptor pairing by infected B cells in contact with cytotoxic T cells, respectively, may trigger NF-κB1 activation *via* the 2B4-binding partner SAP. Loss of SAP or its function has been similarly reported with EBV disease manifestation (62). Future identification of further immunodeficiency-causing genes related in these processes might shed additional light on NF-κB1-related mechanisms in EBV-directed cytotoxic T-cell response.

### Combined Immunodeficiencies Associated with NF-κB Pathways

Among the immediate NF-κB pathway-associated immunodeficiencies, NF-κB1 haploinsufficiency itself has been reported with EBV-driven lymphoproliferative disease or recurrent EBV infection (7, 8, 13). In addition, a recent study reported a patient with a constitutively active mutant of NF-κB2 and EBV viremia (14). NF-κB-related immunodeficiencies harbor the potential to develop EBV-driven disease in a predisposing environment, and thus should be considered at risk. The hitherto identified deficiencies of upstream components of NF-κB have yet not been reported with EBV-related disease. These include homozygous deficiencies of IKK2, NIK, RELB, and X-linked deficiency of NEMO (see Figure 1). NEMO mutations were reported with susceptibility to pyrogenic and myco-bacterial infections, due to specific defects of NK-cell cytotoxicity. Selected affected patients presented with viral infections of molluscum contagiosum or human papillomavirus (73). Homozygous loss-of-function affecting NIK has been shown to affect various B cell compartments, with additional perturbation of NK cell and memory/follicular helper T-cell activity, but lacking evidence of EBV-linked episodes (74). Biallelic loss of *RELB* resulted in a CID with unresponsive T cells and impaired humoral immunity, presenting with respiratory infections but no report of EBV manifestation (75). Both NIK and RelB are crucial players in non-canonical NF-κB2-mediated signaling. NEMO deficiency was reported with severe CID-like characteristics including impaired B- and T-cell activation response through various stimuli, and generally absent regulatory and gamma-delta T cells. Alongside multi-pathogenic infections, viral infections were only reported for parainfluenza type 1 (76, 77).

Lastly, with the CD40/CD40L signaling system, an NF-κB-related mechanism itself is of relevance for successful EBV infection in B cells. CD40 expression by B cells and its engagement with its receptor CD40L on the surface of T lymphocytes is involved in the formation of memory B cells and Ig isotype switching (78). Homozygous mutations affecting either CD40 or CD40L have been shown to cause hyper-IgM syndrome (79–81). In EBV-infected B lymphocytes, CD40/CD40L signaling is induced by a mimicking mechanism, and critical for B-cell transformation (55).

### Molecular Considerations of EBV-Driven Disease in NF-κB1 Haploinsufficiency

The persistent exposure to EBV that is present in over 90% of the human population causes a considerable threat to dysfunctional NF-κB signaling systems. Accumulating evidence enabled by studies on CD27, CD70, and SAP deficiencies points to NF-κB1 being involved in T-cell intrinsic mechanisms downstream of their recognition of EBV-infected B cells (see Figure 3). Due to the only recent discovery of EBV disease and T-cell dysfunctions in NF-κB1 haploinsufficiency, comprehensive studies of T-cell functionalities have not been systematically explored. For example, the T-cell related target gene expression sets modified by the various identified NF-κB1 haploinsufficient conditions should be addressed in a comparative manner. Similarly, it would be necessary to explore detailed T-cell responses, including cytotoxicity and proliferation studies in larger cohorts, to evaluate genotype–phenotype relationships.

Numerous target genes have been found differentially expressed by the NF-κB signaling systems and summarized in their whole on a web resource at Boston University (http://www.bu.edu/nf-kb/gene-resources/target-genes/). Collectively, such affected targets include cytokines and chemokines, T-cell receptor and toll-like receptor subunits, T-cell activation and differentiation markers, cell adhesion molecules, stress response factors, growth factors and regulators of apoptosis, and numerous transcription factors and signaling molecules. Expression profiling would need to clarify which of these could contribute to T-cell-related EBV disease in NF-κB1 haploinsufficiency.

NF-κB signaling itself is critically relevant for EBV replication in B cells. EBV infects B cells by binding of glycoprotein gp350 and upregulation of latency genes such as LMP1 that mimics CD40 and B-cell receptor signaling (55). Subsequent induction of NF-κB signaling upregulates the B-cell reproductive machinery. Still, partial loss of the NF-κB signaling system through NF-κB1 haploinsufficiency has been shown associated with severe EBV proliferative disease (7). Due to the monoallelic nature of the disease, haploinsufficient NF-κB1 signaling might be sufficient for EBV to successfully reproduce in B cells, while at the same time the dysfunctional T cells are rendered unable to clear such infection. In addition, in case of disrupted canonical NF-κB1 signaling, the remaining NF-κB2 axis could sufficiently induce (infected) B-cell replication. EBV protein LMP1 has been shown to activate both the canonical and non-canonical NF-κB signaling networks by using specific domains termed TES1 and TES2, respectively (82). A recent report describes a patient with a heterozygous NF-κB2 precursor-skipping mutation that resulted in a constitutive presence of p52. The mutation was shown to cause CID with severe EBV infection (14). Through pathway crosstalk, NF-κB2 can inhibit NF-κB1 activation by direct interaction of respective subunits (82), and thus potentially lead to similar functional NF-κB1 defects.

### Treatment Considerations for NF-κB- and EBV-Related Immune Deficiencies

Lymphoproliferation by EBV infection causes life-threatening autoimmune-like infiltration into target organs that demands aggressive treatment. Given this pressing urgency, the fatal correlation of EBV-associated disease occurring predominantly in immunodeficient individuals causes a discrepancy in the choice of treatment—simply spoken, a fully satisfactory therapy recommendation is still elusive. Virus-induced lymphocyte expansion is tackled by application of various immunosuppressants. Yet the use of immune-suppressing agents themselves additionally dampens the genetically compromised immune cell function. EBV infection and/or reactivation can be managed by combined application of rituximab and corticosteroids for depletion of (infected) B cells and infiltrating T cells (7). Allogeneic hematopoietic stem cell transplantation (aHSCT) should be considered in severe cases with recurring EBV disease (7). Still, such measures often fail to protect against viral infections (55).

In addition to unspecific B-cell depletion with CD20- or CD19-directed monoclonal antibodies, EBV-T cell-specific antibody therapy against viral proteins such as LMP1 is currently being discussed (52). Boosting immunity against EBV is similarly considered (54). This includes vaccination with the viral gp350 glycoprotein, or an elaborate T-cell boost by *in vitro* expansion of virus-specific T cells followed by re-infusion into the patient’s circulation (54). In addition, administration of interleukin-7 was reported to expand virus-specific cytotoxic T cells (83).

Targeting virus expansion directly at its core would manage EBV-driven autoimmunity, especially when administered in combination with rituximab or other eliminators of infected B cells (54). Currently available antiviral therapeutics such as acyclovir only target the lytic/replicative state of viral infections, leaving the latently infected cells intact for breakout at a later stage (52, 54). Inhibitors of viral proteins such as LMP1 might prove beneficial in future studies, but are still in developmental stage. Drugs acting downstream of the EBV pathway, including PI3K, AKT and MAPK inhibitors, are in turn not specific in targeting only the virus-carrying host cells. Proteasome inhibitors are similarly non-specific. Zou and coworkers reported that bortezomib induced apoptosis in EBV-transformed B cells by interfering with NF-κB signals, prolonging survival in mice (84).

Proteasome inhibitors are as well being discussed in context of NF-κB deficiencies (85) and might hence be worth investigating in specific NF-κB1 disorders, especially in combination with EBV-induced disease. NF-κB1 dysfunction as consequence of accelerated degradation of mutated protein might be tackled by blocking the proteasome with specific inhibitors. On the other hand, proteasome inhibitors could also hinder IκB clearance and in turn inhibit NF-κB function, hence a dedicated study of affected NF-κB1 haploinsufficiency would be necessary to investigate a possible outcome. Drugs targeting the transcription machinery to restore or enhance *NFKB1* transcription, or to block IκB expression, are currently being discussed at a research level, though raise the concern of specificity. Targeted degradation of IκB seems an attractive novel approach. The recent advances in the development of degradation-directing therapeutics (86) might prove relevant for NF-κB-related disorders. When considering therapeutic induction or stabilization of NF-κB, it needs to be clarified to which extent excessively active NF-κB could increase the risk for hematological malignancies (87). Similar concerns apply for general kinase activators.

Among the reported drugs to interfere with NF-κB pathways, the chemotherapeutic etoposide has been reported to increase NF-κB (85, 88). Intriguingly, etoposide is part of the current HLH2004 protocol for treating hemophagocytic lymphohistiocytosis, a disease that is often triggered by EBV infection (89). In light of chemotherapy being considered for EBV-driven disease (52), a combined rationale in case of NF-κB dysfunction might prove beneficial. On the other hand, numerous agents have been shown to downregulate NF-κB activation by various mechanisms and are summarized by Yamamoto and Gaynor (85). These include anti-inflammatory steroids and glucocorticoids, such as prednisone and dexamethasone, as well as non-steroidal anti-inflammatory drugs, such as aspirin and sodium salicylate. Similarly, the common immunosuppressants cyclosporine A and tacrolimus were reported to inhibit calcineurin and, thus, NF-κB pathways (85), although calcineurin can also exhibit positive modulatory activity for NF-κB (90).

On a larger scale, whether general or specific indications for invasive aHSCT are being met is only starting to be discussed in expert communities. Severe or recurrent disease manifestation with EBV-lymphoproliferative disease may represent an indication for such invasive treatment strategy, though owing to the lack of (published) data, it is currently not clear what the outcome of aHSCT in NF-κB1-mutant PID is with regard to long-term morbidity and survival. To define improved guidelines and assistance in therapeutic decisions, it will be necessary in the upcoming years to collect comprehensive data revealing phenotype–genotype relationships and long-term surveillance of this rather novel disease.

Altogether, EBV infectious disease and chronic EBV infections as a consequence of NF-κB1 (7, 8, 13) and also NF-κB2 (14) dysfunction deserve special consideration. Though initiated in early stages, personalized approaches for immune deficiencies with viral predisposition remain a challenge that will direct future considerations regarding cellular, genetic, immune, and drug therapies. The continuing urgent demand for virus-targeting therapeutics, and the increasing emergence of NF-κB-related immunodeficiencies within the past and coming years, will enable new lines of discussions.

## Conclusion

Among the primary immunodeficiencies, haploinsufficiencies are considered inherently diverse in disease manifestation and penetrance (91). Similarly, CVIDs have been grouped based on a very heterogeneous collection of diseases (40–42). Monoallelic mutations in *NFKB1* causing genetic or functional NF-κB1 haploinsufficiency have recently been reported to not only account for CVID-like B-cell deficiency, but a rather complex immunodeficiency-like profile including the emergence of combined B- and T-cell dysfunction (5–13). As a consequence of affected T-cell integrity, NF-κB1 haploinsufficiency has been shown to coincide with recurrent EBV and life-threatening EBV-driven lymphoproliferative disease (7, 8, 13). The latter poses a challenge due to immediate urge and yet often unsatisfactory treatment options in immunodeficient individuals. Especially when facing a disease with broad clinical manifestation and highly individual genotype–phenotype presentation, defining tailor-made treatment options will be highly relevant. Based on the currently available clinical and immunological data, the causative relation between defined *NFKB1* mutations and EBV infections or associated disease remains unclarified. Larger cohorts and a broad investigation of cellular and genetic functionalities will be necessary to decipher this relationship. Comparison of EBV-associated mechanisms of immunodeficiencies and the NF-κB signaling system suggests that dysfunctional T-cell core processes underlie a particular vulnerability to EBV infection. It will, thus, be of urgent relevance to further investigate T-cell functions affected by NF-κB1 haploinsufficiencies, in order to understand this highly heterogeneous disorder and its relevance to EBV-associated lymphoproliferative disease.

## Author Contributions

BH performed literature research, designed the layout, and wrote the manuscript. NS performed literature research and wrote the manuscript. KB coordinated the study, performed literature research, and wrote the manuscript.

## Conflict of Interest Statement

The authors declare that the research was conducted in the absence of any commercial or financial relationships that could be construed as a potential conflict of interest.

## Abbreviations

aHSCT, allogeneic hematopoietic stem cell transplantation; AKT, AKT serine/threonine kinase; ATM, ataxia telangiectasia mutated serine/threonine kinase; BAFF(-R), B-cell activating factor (receptor); BCL10, B-cell CLL/lymphoma 10; BCR, B-cell receptor; BIRC4, Baculoviral IAP-repeat containing protein 4; CARD11, caspase recruitment domain family member 11; CARMA, CARD-containing MAGUK protein 1; CD40L, CD40 ligand; CID, combined immunodeficiency; CTPS1, CTP synthase 1; CORO1A, Coronin 1A; CVID, common variable immunodeficiency; DD, death domain; EBV, Epstein–Barr virus; FCGR3A, Fc fragment of IgG receptor 3A; GATA2, GATA binding protein 2; GRR, glycine-rich region; IκB, inhibitor of NF-κB; IKK, inhibitor of NF-κB kinase; IL-1, interleukin-1; IL1R, interleukin-1 receptor; IRAK, interleukin-1 receptor-associated kinase 1; ITK, IL2-inducible T-cell kinase; LMP1, latent membrane protein 1; LPD, lymphoproliferative disease; LPS, lipopolysaccharide; LTβ(R), lymphotoxin β (receptor); MAGT1, magnesium transporter 1; MALT1, mucosa-associated lymphoid tissue lymphoma translocation protein 1; MAPK, mitogen-activated protein kinase; MCM4, minichromosome maintenance protein 4; NEMO, NF-κB essential modulator; NF-κB1/NF-κB2, nuclear factor kappa-light-chain-enhancer of activated B cells-1/-2; NIK, NF-κB inducing kinase; NLS, nuclear localization sequence; PID, primary immunodeficiency; PI3K, phosphatidylinositol 3-kinase; PIK3CD, phosphatidylinositol 3-kinase catalytic delta; PIK3R1, phosphatidylinositol 3-kinase receptor 1; PKC, protein kinase C; RASGRP1, rat-sarcoma guanyl releasing protein 1; RHD, rel-homology domain; SAP, signaling lymphocytic activation molecule-associated protein; *S. pneumoniae, Streptococcus pneumoniae*; STK4, serine/threonine protein kinase 4; TCR, T-cell receptor; TES1/TES2, transformation effector site 1/2; TIR, toll-like/interleukin-1 receptor; TLR, toll-like receptor; TRIF, TIR-domain containing adaptor-inducing interferon-β; TNFα, tumor necrosis factor-α; TNFR, tumor necrosis factor receptor; TRADD, tumor necrosis factor receptor type 1-associated DEATH domain protein; TRAF, tumor necrosis factor receptor-associated factor; WASP, Wiskott–Aldrich syndrome protein.
